# A Unique Case of Granulomatosis With Polyangiitis With Cutaneous Manifestations Developing a Decade Later

**DOI:** 10.7759/cureus.103988

**Published:** 2026-02-20

**Authors:** Sophia E Kujawski, Betty Hsiao

**Affiliations:** 1 Rheumatology, Yale School of Medicine, New Haven, USA

**Keywords:** anca-associated vasculitis, granulomatosis with polyangiitis (gpa), palpable purpura, petechiae, pyoderma gangrenosum-like lesions, rash

## Abstract

We present the case of a 60-year-old man with granulomatosis with polyangiitis (GPA). While he initially presented with only renal involvement, he developed cutaneous manifestations more than a decade later. Cutaneous manifestations may manifest at any point during the duration of the disease, although most usually present at the onset. Cutaneous manifestations of GPA include petechial-like lesions and palpable purpura, typical of what is seen with small vessel vasculitides, as well as pyoderma gangrenosum (PG)-like lesions, digital necrosis, subcutaneous nodules, and/or livedo, which can also be seen but may be more commonly associated with medium vasculitis. As these manifestations may also be found in tandem with other diseases, it is important to include GPA as part of the differential diagnosis when such skin lesions are identified. Treatment of cutaneous manifestations of GPA includes glucocorticoids and immunosuppressive agents.

## Introduction

Granulomatosis with polyangiitis (GPA) is a rare autoimmune small vessel vasculitis that leads to the development of necrotizing granulomas and can often be fatal if left untreated [1]. GPA is the most common of the three antineutrophil cytoplasmic antibody (ANCA)-associated vasculitides, with microscopic polyangiitis (MPA) and eosinophilic granulomatosis with polyangiitis (EGPA) occurring significantly less frequently. GPA occurs primarily in adults, affecting about 120-140 per million in populations of American and European ancestry. GPA is reported equally between men and women [1].

The exact pathogenesis is unknown but thought to involve anti-neutrophilic cytoplasmic autoantibodies (ANCA); additionally, interactions between genetics, the immune system, and infectious organisms have also been implicated. While GPA preferentially affects the upper respiratory tract and pulmonary and renal systems, manifestations can be found in almost any organ, including the skin, eyes, and joints [1].

## Case presentation

A 60-year-old man who underwent gastric bypass in April 2008 with a postoperative course complicated by bowel perforation and sepsis, intubation followed by tracheostomy, and then decannulation, was discharged to a rehabilitation center when he developed acute renal failure with hematuria in October 2008. His baseline serum creatinine rose from 1.2 mg/dL to 3.7 mg/dL when he was readmitted to the hospital, and he was found to have positive perinuclear antineutrophil cytoplasmic antibody (P-ANCA) (Table 1) with anti-proteinase 3 antibody (PR3) and anti-myeloperoxidase (MPO) antibody status unknown.

**Table 1:** Laboratory results ANCA: antineutrophil cytoplasmic antibody, P-ANCA: perinuclear antineutrophil cytoplasmic antibody

Laboratory results	Result	References ranges
ANCA screen	P-ANCA positive	Negative
Serum creatinine (09/07/2008)	1.2 mg/dL	0.72-1.35 mg/dL
Serum creatinine (10/07/2008)	3.7 mg/dL	0.72-1.35 mg/dL
Serum creatinine (12/11/2008)	1.4 mg/dL	0.72-1.35 mg/dL

He underwent a renal biopsy that showed pauci-immune crescentic nephritis and was diagnosed with GPA based on pathology and serologies (images of histology are not available). He was treated with high-dose intravenous (IV) glucocorticoids (methylprednisolone 1 gram IV daily for three days and then transitioned to prednisone that was tapered over six months) and intravenous cyclophosphamide as induction therapy. He was followed by nephrology and rheumatology subspecialties after his initial diagnosis; while his renal function did not return to his baseline of serum creatinine 1.2 mg/dL, his renal function remained stable as stage III chronic kidney disease (CKD) with serum creatinine 1.4 mg/dL without need for renal replacement or maintenance immunosuppression (Table 1).

**Figure 1:**
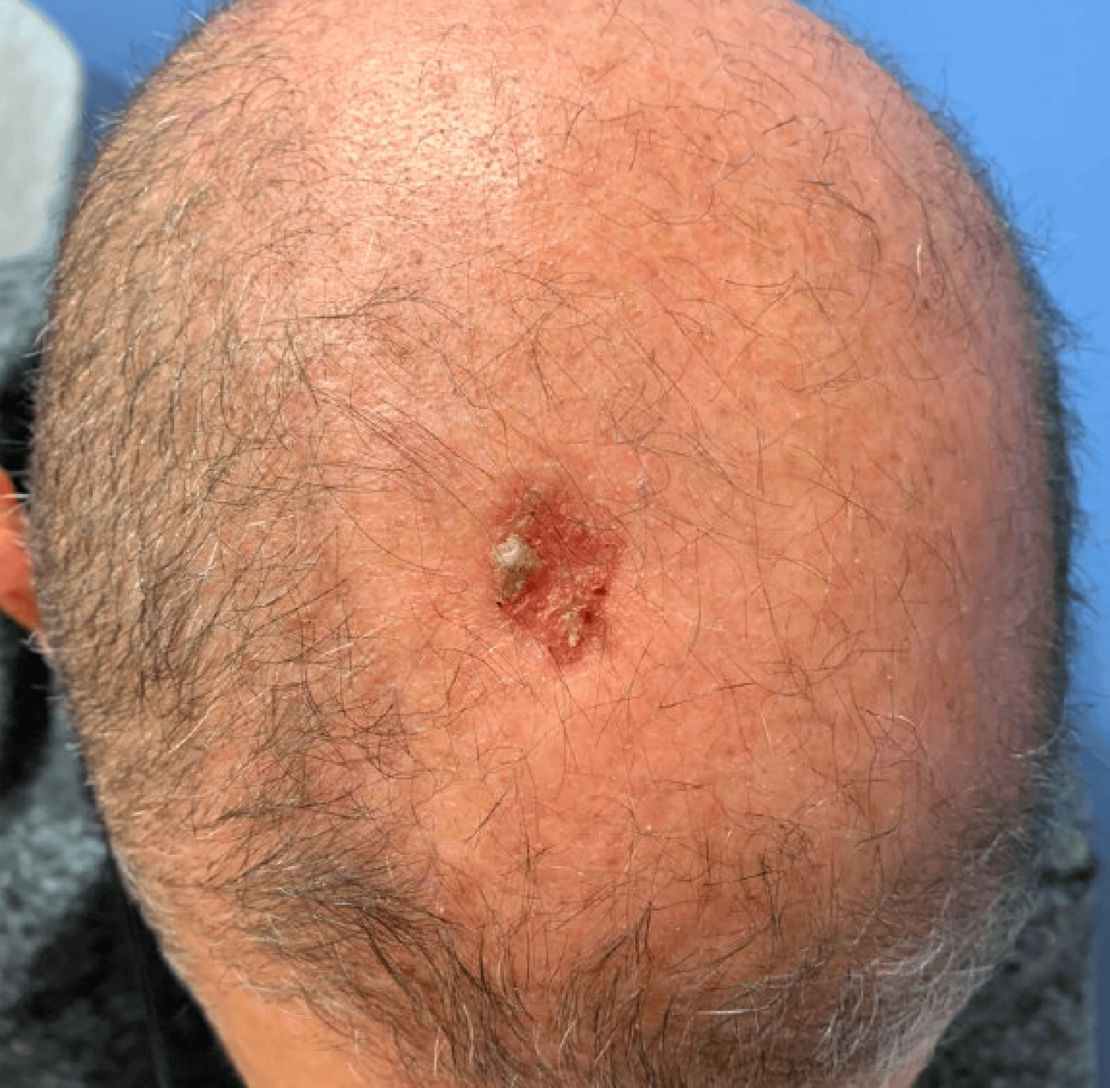
Photograph of the scalp lesion prior to treatment

**Figure 2:**
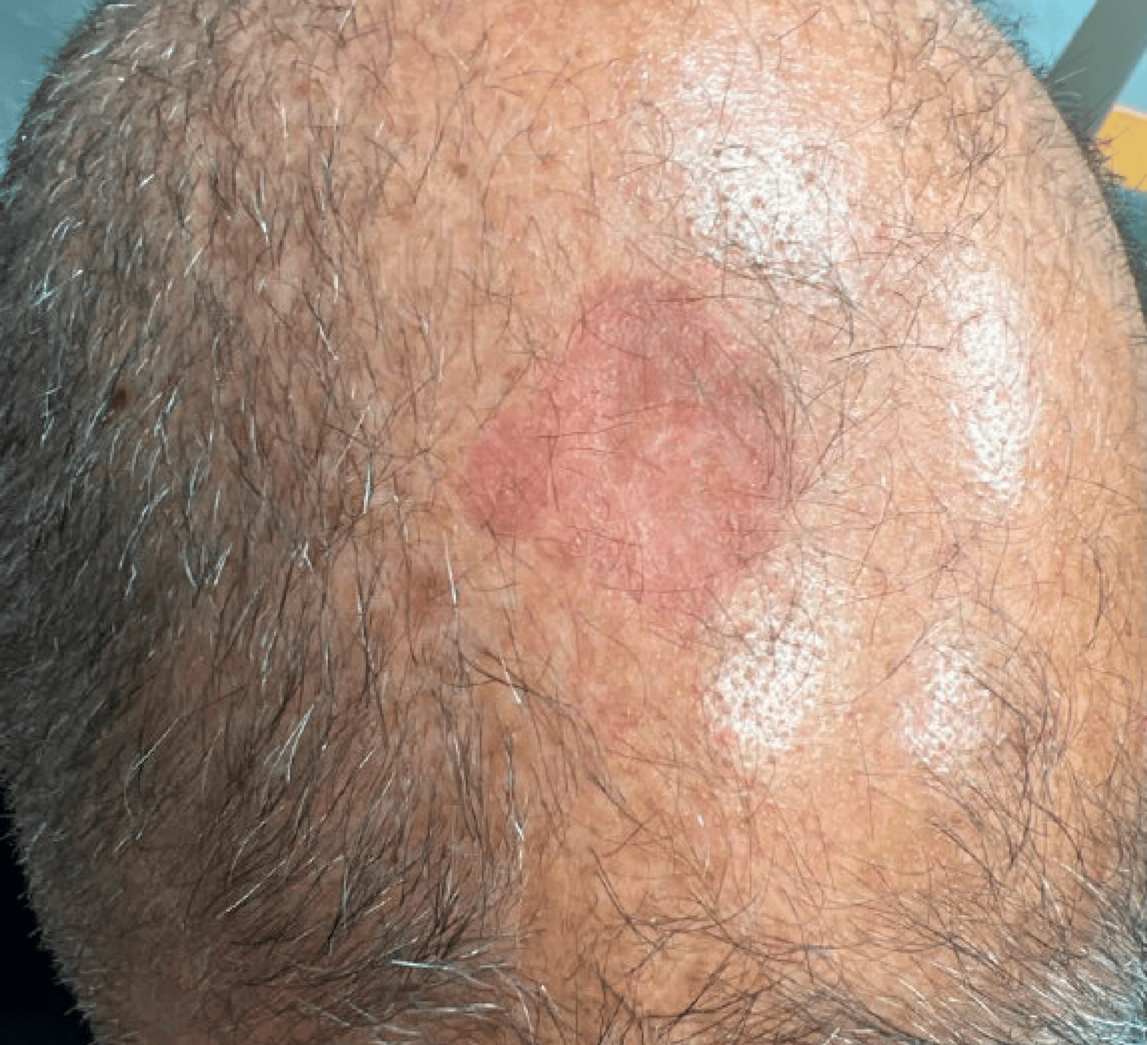
Photograph of the scalp lesion after treatment

While he did not have sinus or lung involvement at the time of initial presentation, he later developed sinus congestion and epistaxis in late 2009, leading to chronic anosmia and ageusia. He was evaluated by multiple otolaryngologists and underwent endoscopic sinus surgery. He was treated with prednisone tapers (starting at 30 mg/day tapered over six days) with only temporary improvement in taste and smell. He was also trialed on oral methotrexate as a steroid-sparing agent (with a maximum dose of 15 mg weekly) without efficacy. He then developed pruritic, raised, crusted lesions on his face and scalp 14 years after initial diagnosis in 2022 (Figure 1), with biopsy of right scalp lesion that showed crusted and impetiginized granulomatous dermatitis thought to be related to underlying GPA (Figure 2).

**Figure 3:**
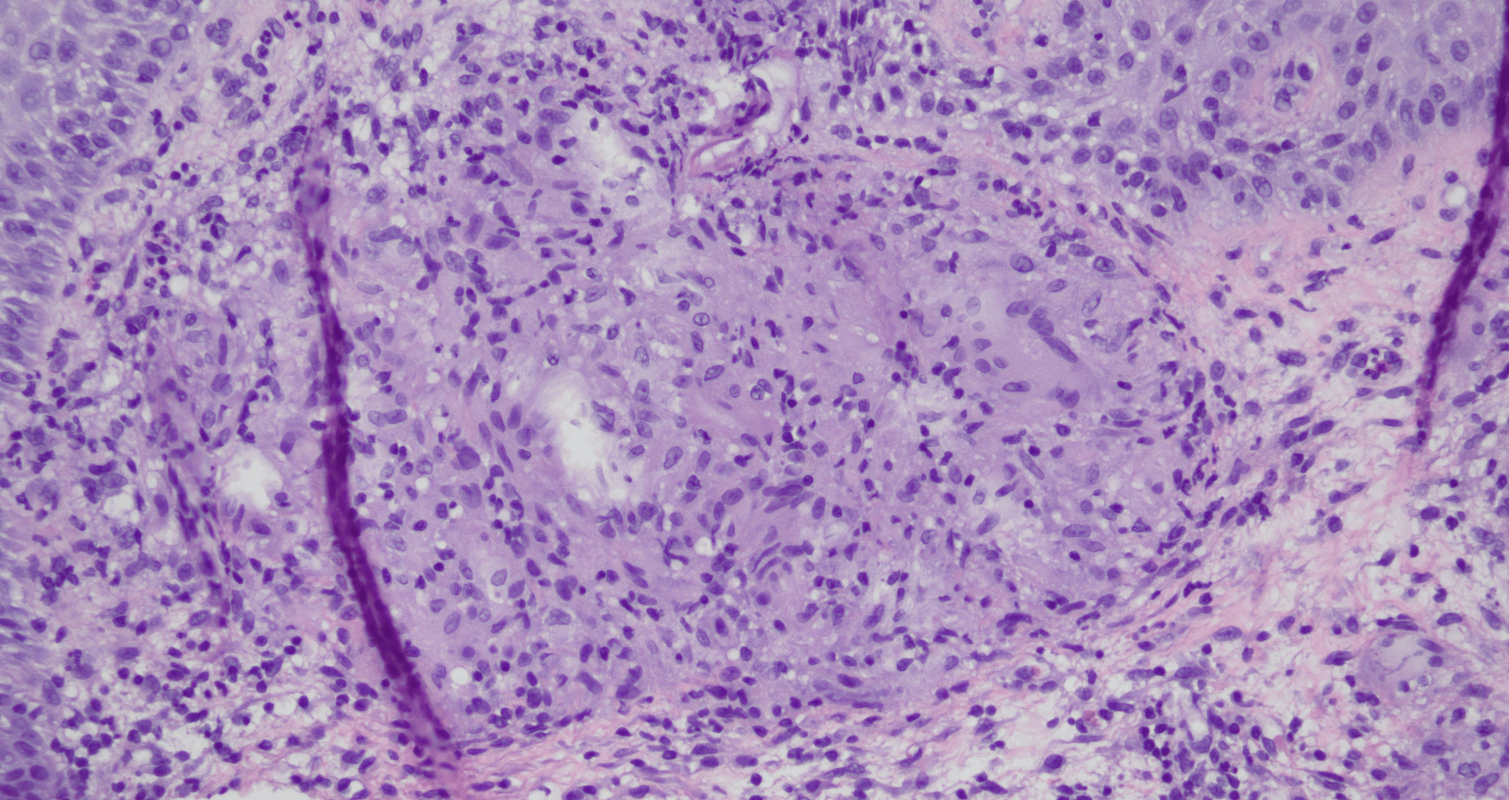
Dermatopathology of biopsy from the right central scalp in formalin Pathology showed granulomatous dermatitis with PAS, Fite, acid-fast, and Gram stains negative for organisms. Gross description: received in formalin Dimensions: 7.5 x 4 x 1 mm, bisected and submitted in one cassette PAS: periodic acid-Schiff

**Figure 4:**
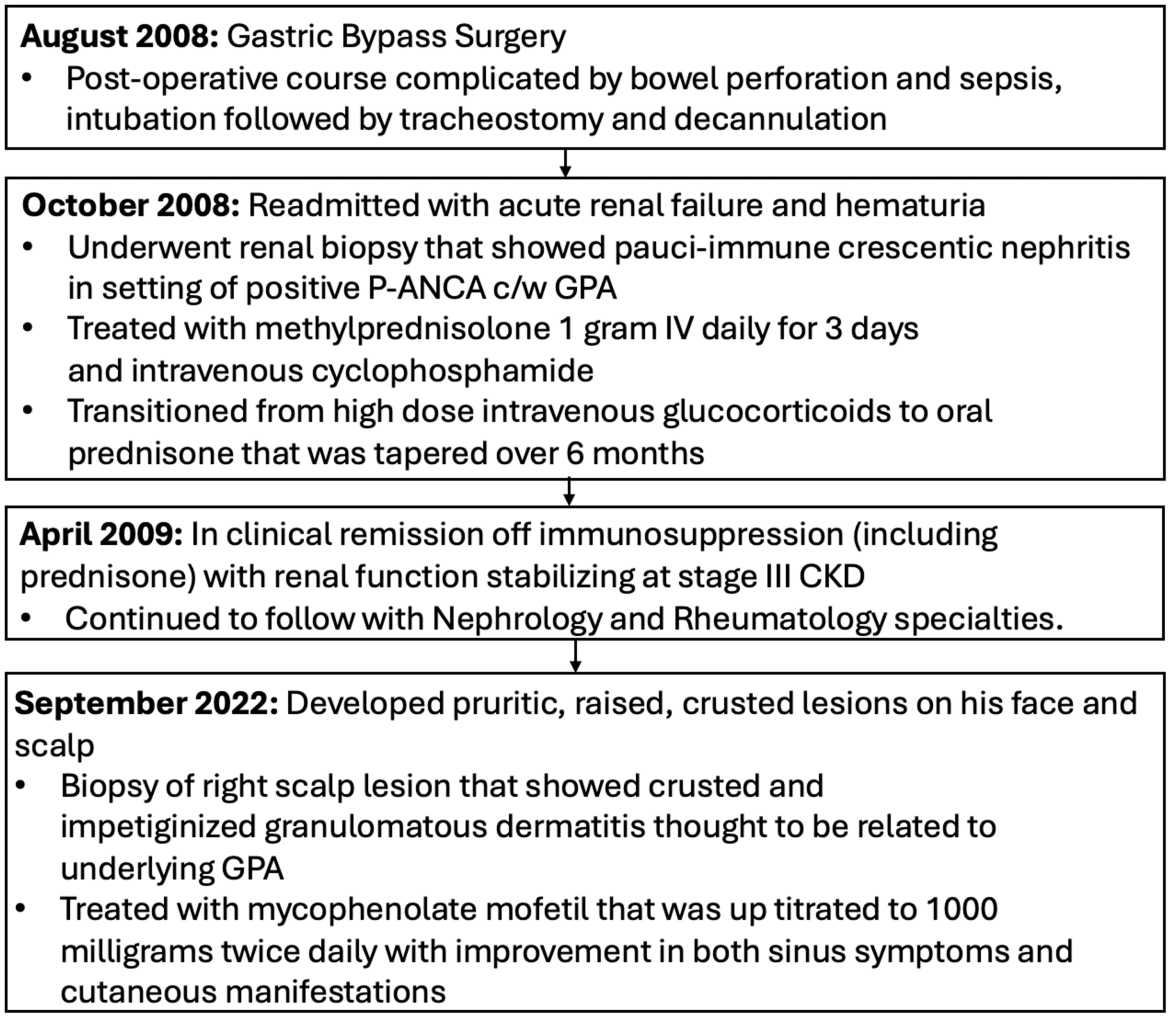
Patient disease course P-ANCA: perinuclear antineutrophil cytoplasmic antibody, GPA: granulomatosis with polyangiitis, CKD: chronic kidney disease

Repeat serologies were notable for negative ANCA, PR3, and MPO antibodies (Figure 3). He was started on mycophenolate mofetil that was uptitrated to 1,000 mg twice daily, with improvement in both sinus symptoms and cutaneous manifestations. His disease course is summarized in Figure 4.

## Discussion

Cutaneous manifestations have been reported in up to 35%-50% of patients with GPA [2]. These manifestations may occur at the onset of the disease or at any time point throughout the disease course [3]. There are several types of associated cutaneous manifestations, which can be found in isolation or in combination with each other [4]. Cutaneous manifestations are not contagious to others but may lead to secondary infections and further complications in terms of wound healing.

The most common cutaneous manifestation of GPA is petechial-like lesions, which are small red spots caused by dermal hemorrhage, frequently found on the lower limbs [5]. These lesions can provide a helpful clue to a vasculitis diagnosis as they are easily identifiable on a physical examination, and histological patterns of leukocytoclastic vasculitis (LCV) can be found in biopsies stained with hematoxylin and eosin [6]. Typical histological findings of LCV include “upper dermal perivascular infiltrates mainly composed of neutrophils with karyorrhexis of nuclei, swelling of the endothelium, fibrinoid necrosis of vessel walls, and extravasation of erythrocytes.”

Palpable purpura, similar to petechiae-like lesions, results from broken capillaries under the skin [4]. Purpura is characterized by large, fluid-filled, raised blisters on the extremities [6] and may present in the form of purpuric macules, papules, and ecchymosis plaques [4]. Purpura has been reported in up to 38% of cutaneous manifestations in some case series and is also readily identifiable as non-blanching erythematous lesions on physical examination [2]. Biopsies of affected sites yield identification of leukocytoclastic vasculitis affecting dermal small vessels [6]. Immunofluorescence often shows immunoglobulin M deposits in small vessels. Complement C3 perivascular deposits have been reported in up to 80% of purpura biopsies [6].

Pyoderma gangrenosum (PG)-like lesions may initially appear as small, red pustules that may evolve into large, open, painful ulcers [4]. These lesions have a similar semblance but lack specific characteristics of pyoderma gangrenosum, such as a raised, undetermined border and predominantly neutrophilic infiltrate on histology [4]. More than half of patients with PG develop cutaneous lesions in association with an underlying systemic disease, most commonly inflammatory arthritis, inflammatory bowel disease, or a hematologic disorder. Differential diagnoses include vascular disorders, infection, malignancy, drugs, and thrombotic disorders.

Digital necrosis may occur in up to 10% of GPA cases that often involve decreased vascular perfusion from active vasculitis [2]. Initial symptoms may include pain and swelling, followed by gangrene if not treated. Physical examination features include duskiness in the digits, along with ischemic changes such as necrosis. Histological features of necrosed digits often include necrotized vessel walls with arterial thrombi and are often nonspecific, so diagnosis should be made in tandem with other GPA-specific manifestations.

Subcutaneous nodules may present on the lower limbs and have been reported in less than 15% of patients with GPA [4]. The pathophysiology involves inflammation of medium-sized vessels in the deep dermis and subcutis, leading to nontender, firm nodules that may ulcerate. Histology shows diffuse infiltration of inflammatory cells around blood vessels.

Livedo reticularis presents as skin discoloration in a lacy, net-like pattern from involvement of small- and medium-sized vessels in the extremities, often occurring alongside nodules and ulcers on the lower extremities. This manifestation is commonly found in other autoimmune conditions such as systemic lupus erythematosus, systemic sclerosis, and Sjögren’s syndrome. Livedo racemosa can also be seen in patients with GPA, exhibiting a larger, broken pattern as compared to livedo reticularis. Livedo racemosa stems from permanent loss of peripheral blood flow and does not reverse on warming.

Table 2 describes the types of cutaneous manifestations [7-11].

**Table 2:** Cutaneous manifestation types PG: pyoderma gangrenosum Adapted from references [7-11]

Cutaneous manifestation type	Description	Histology
Petechiae	Small red spots caused by dermal hemorrhage, frequently found on the lower limbs	Pauci-inflammatory thrombogenic vasculopathy with extensive deposition of complement components within the cutaneous microvasculature are seen in pathological view [7]
Palpable purpura	Large, fluid-filled raised blisters on the extremities	Palpable purpuras show cellular infiltration into the obscured small vessel walls with infiltration of polymorphonuclear cells [7]
PG-like lesions	May initially appear as small, red pustules that may evolve into large, open, painful ulcers	Infiltrate of neutrophils in the dermis, may contain necrotic dermal vessels, direct immunofluorescence is nonspecific [8]
Digital necrosis	Duskiness in the digits, along with ischemic changes such as necrosis	Necrotized vessel walls with arterial thrombi [7]
Subcutaneous nodules	Involves inflammation of medium-sized vessels in the deep dermis and subcutis, leading to nontender, firm nodules that may ulcerate	Diffuse infiltration of inflammatory cells around blood vessels [9]
Livedo reticularis	Skin discoloration in a lacy, net-like pattern from involvement of small- and medium-sized vessels in the extremities	Pauci-inflammatory microthrombotic vasculopathy [10] and perivascular lymphocytic infiltration are seen on pathological examination [11]; complement deposition within and around vessels has also been described

Early diagnosis and prompt treatment of GPA are critical in improving outcomes and decreasing mortality [2]. The most common initial treatment for GPA is induction immunosuppression with a regimen of glucocorticoids (normally high dose, followed by a taper) along with intravenous anti-CD20 monoclonal antibody therapies, which has become the gold standard of treatment [2]. Treatment is usually tailored to the severity of the disease. Maintenance immunosuppression can include oral agents such as methotrexate, azathioprine, mycophenolate mofetil, and complement 5a receptor antagonist avacopan, as well as intravenous agents such as anti-CD20 monoclonal antibody therapies and cyclophosphamide. Typically, cutaneous manifestations can be initially treated with targeted oral or topical treatments such as triamcinolone, hydrocortisone, or betamethasone. These topical agents, along with antihistamines, can reduce the symptoms of burning or itching [6].

## Conclusions

We present the case of a 60-year-old man who initially presented with acute renal failure with hematuria in the setting of positive P-ANCA, with renal biopsy revealing paci-immune crescentic nephritis, consistent with a diagnosis of GPA. He was successfully treated with glucocorticoids and cyclophosphamide, although his renal function did not return to baseline and stabilized at stage III CKD without the need for renal replacement therapy.

Although his initial presentation was renal-limited GPA, our patient developed sinus manifestations the year following his diagnosis. His symptoms of sinus congestion and epistaxis led to chronic anosmia and ageusia that persisted despite treatments that included endoscopic sinus surgery, glucocorticoid tapers, and oral methotrexate. More than a decade after his initial diagnosis, the patient then developed raised and crusted lesions on his face and scalp, with a biopsy that showed findings consistent with impetiginized granulomatous dermatitis thought to be related to his underlying GPA. He was started on mycophenolate mofetil with significant improvement in his cutaneous and sinus manifestations.
